# Connecting with healthcare providers at diagnosis: adolescent/young adult cancer survivors’ perspectives

**DOI:** 10.1080/17482631.2017.1325699

**Published:** 2017-06-15

**Authors:** Celeste R. Phillips, Joan E. Haase, Marion E. Broome, Janet S. Carpenter, Richard M. Frankel

**Affiliations:** ^a^ Department of Science Nursing Care, Indiana University School of Nursing, Indianapolis, IN, USA; ^b^ Division of Healthcare of Women and Children, Duke University School of Nursing, Durham, North Carolina, United States; ^c^ Department of Internal Medicine, Indiana University, School of Medicine Indianapolis, IN United States

**Keywords:** Adolescents, young adults, connectedness, communication, cancer, survivors

## Abstract

Adolescents and young adults (AYAs) with cancer are a vulnerable and underserved population. AYAs’ cancer survivorship is complicated by physical and psychosocial late effects which requires long-term follow-up. Connectedness with healthcare providers (HCPs) is a protective factor that may improve long-term follow-up behaviours of AYAs. However, little is known about AYAs’ experiences connecting with HCPs. The purpose of this study was to describe AYA cancer survivors’ experiences connecting with HCPs. This empirical phenomenological study interviewed nine AYA cancer survivors diagnosed during adolescence. Individual interviews were conducted and analysed using an adapted Colaizzi approach. The essential structure reveals that AYAs begin their experience of connectedness with a sense of disconnectedness prior to treatment. The diagnosis is a period of confusion and emotional turmoil that interfere with the AYAs’ ability to connect. When AYAs come to accept their illness and gain familiarity with the environment, they then put forth an effort to connect with HCPs. Although it takes time for AYAs to reciprocate efforts to connect, HCPs should be aware that AYAs carefully assess and make judgments about whether or not HCPs can be trusted. Findings raise awareness of the actions and behaviours of HCPs that hinder connectedness, and targeted in future research.

## Introduction

Adolescents and young adults (AYAs) (ages 15–29 years) with cancer have shown strikingly less improvement in treatment outcomes than either younger or older cancer patients, even though there has been progress in the treatment of childhood cancer over the past four decades (Bleyer et al., 2017). Adolescents and young adults have higher mortality and lower 5-year survival rates than younger children (Bleyer et al., 2017). Importantly, young adult cancer survivors who were diagnosed and treated for cancer during adolescence have poorer psychosocial outcomes than other age groups and are considered to be a vulnerable population (Institute of Medicine (IOM), 2013); Nass et al, 2015.

AYAs are at risk for developing adverse health problems secondary to their previous cancer therapy. Treatment-related complications, also known as “late effects”, include neurocognitive dysfunction, cardiopulmonary toxicity, endocrinopathy, psychological difficulties and secondary malignancies (Adolescent and Young Adult Oncology Progress Review Group (AYAO PRG), 2006). Researchers estimate that as many as two-thirds of young adult cancer survivors have at least one late effect, with about one-third having serious or life-threatening complications (Oeffinger & Wallace, 2006). Many of these late effects remain dormant for decades and require careful screening and monitoring throughout life. However, recent studies have indicated that the cancer screening behaviours and medical follow-up practices of AYA cancer survivors are less than optimal (Oeffinger et al., 2004; Oeffinger & Wallace, 2006).

In addition to late effects, there is evidence that AYA cancer survivors engage in lifestyle behaviours that are likely to further increase their risk of subsequent cancer and other chronic illnesses. Some of these risk behaviours include substance abuse (e.g., alcohol, cigarettes and drugs), insufficient physical activity, non-adherence to sun-protection recommendations and suicide attempts (Phillips-Salimi, Lommel, & Andrykowski, 2012; Recklitis, Lockwood, Rothwell, & Diller, 2006; Tai et al., 2012). Although there is mixed evidence regarding the prevalence of these risk-taking behaviours, even low rates are alarming due to the survivors’ risk of developing late effects. Thus, there is a need to identify protective factors that will help AYA cancer survivors monitor potential late effects, adopt better health behaviours and ultimately improve their overall health and well-being.

Connectedness with healthcare providers (HCPs) is a potential protective factor that may diminish risk-taking behaviours and promote resilience and enhanced well-being in AYAs (Haase, 2004). Maintaining a supportive, positive relationship HCPs throughout survivorship is believed to be associated with the engagement in cancer screening, decision-making and healthcare management during treatment and survivorship. Research with adult patients indicates patients’ perceptions of connectedness (i.e., having a close, meaningful relationship) with HCPs is associated with increased participation in decision-making (Leidy & Haase, 1996; Marelich & Murphy, 2003; Sheppard, Adams, Lamdan, & Taylor, 2011), increased treatment adherence (Beach, Keruly, & Moore, 2006; Brion, 2014; Earle, Davies, Greenfield, Ross, & Eiser, 2005), and decreased risk-taking behaviours (Beach et al., 2006; Diaz, Mainous, Gavin, Player, & Wright, 2015). However, little is known about AYAs’ experiences connecting with HCPs.

The purpose of this study was to describe AYA cancer survivors’ experiences connecting with HCPs. Findings presented here are part of a larger phenomenological study aimed at exploring AYAs’ experiences of connectedness with HCPs. Participant experiences were described across the cancer continuum from diagnosis to survivorship. Due to the extensiveness of the meanings and descriptions, we are presenting the findings of the phenomenological study in four separate papers: (1) experiences of connecting with HCPs; (2) experiences of HCPs making the connection with AYAs; (3) experiences of disconnectedness with HCPs; and (4) experiences connectedness during survivorship. This article describes first of these experiences (i.e., the lived experience of AYAs connecting with HCPs). We used a phenomenological research approach that teases out the essential structure that constitutes this phenomenon (Colaizzi, 1978; Husserl, 1970). Such information is essential to understanding the meaning of connectedness to AYAs and how connectedness may influence the long-term health and well-being of AYAs.

## Methods

### Participants

AYA cancer survivors were recruited from a childhood cancer survivor clinic in the state of Indiana in the USA. As this was a phenomenological study aimed at describing the essential structure of connecting with HCPs, a purposive approach was used. Eligibility criteria included: (1) diagnosed with and treated for cancer during adolescence (ages 15–21); (2) currently aged 18–24 years; and (3) completed treatment =1 year and = 9 years ago. Participants were either approached in-person and via telephone to ascertain interest in sharing their experiences of connectedness.

Nine participants aged 20–23 years, diagnosed between ages 15–18 were interviewed. Five were female and four were males. Eight were Caucasian; one was African American. Diagnoses included: osteosarcoma (n = 3); lymphoma (n = 2); ovarian germ cell tumour (n = 2); and leukaemia (n = 2). Participants’ cancer treatments lasted from 3 to 38 months. At the time of interview, the time-since-diagnosis ranged from 1.5 to 5.5 years. Eight participants still had yearly cancer survivor clinic visits; one no longer required follow-up appointments. None of the participants were married and all still lived at home with their parents.

### Data collection

Data were collected by the first author during individual, face-to-face interviews at a time and private location convenient to the participant. Interviews were digitally recorded.

In empirical phenomenology, detailed descriptions of an experience are elicited through a broad data-generating question (Cresswell, 1998). For this study, the data-generating question was:

Please tell me about your experiences of connectedness with healthcare providers. Perhaps you experienced a strong connection with a healthcare provider. Perhaps you perceived yourself as never being connected with a healthcare provider. Or you might have experienced a connection but then became disconnected from your healthcare provider for some reason. Whatever your experiences were, I would like to hear about them. It is sometimes most useful to tell your experiences as a story, starting at the beginning of your contact with healthcare providers. Please describe your experience as fully as you can, including all the circumstances, thoughts, and feelings you can remember.

The question was given to participants at least three days before the interview so they could thoughtfully reflect on their experiences. The goal of each interview was to obtain a rich description of the experience and ensure the participant, not the interviewer, determined the details of the experience discussed (Giorgi, 2005). Open-ended questions and probes were used to encourage a full description of the experience. Interviews lasted between 15 and 99 (*M* = 43.2) minutes.

### Data analysis

Interviews were transcribed verbatim by a transcriptionist, reviewed for accuracy and analysed using an adapted Colaizzi procedure (Colaizzi, 1978; Haase, 1987). Analysis included: (1) listening to interviews several times to gain an understanding of meanings conveyed; (2) identifying significant phrases, restating them in general terms, formulating meanings and validating meanings through research team discussions to reach consensus; (3) identifying and organizing themes into clusters and categories; (4) developing a full description of themes; and (5) describing the essential structure of the experience. Analysis was done collaboratively by two research members (CP and JH) and managed using a combination of Microsoft Word tables and outline features.

### Trustworthiness

Trustworthiness and credibility (Guba & Lincoln, 1981; Sandelowski, 1986) were established in several ways. First, the adapted Colaizzi analysis procedures were systematically applied. Second, analysis was done through research team collaboration and review processes to reach consensus. Results were then reviewed by a panel of three team members who were not involved in the initial analysis. Third, an audit trail was maintained to ensure all analysis steps could be traced back to original interviews.

### Ethical considerations

Institutional Review Board approval was obtained from Indiana University prior to recruitment. All participants were informed about the study in writing and orally before the interview, and informed consent was collected. To protect the participants’ confidentiality, pseudonyms were assigned for participants, HCPs and facilities described in the results below.

## Results

### Essential structure

The essential structure of AYAs’ experiences connecting with HCPs begins when they seek medical advice for unusual symptoms. When answers were not provided in a timely or sensitive matter, their fears and uncertainty about the abnormal findings turn into frustration. The situation is even more upsetting when AYAs are misdiagnosed or misinformed during initial consultations. HCPs who display a lack of sensitivity or respect for AYAs’ concerns exacerbate the difficulties connecting, which leads to a sense of disconnectedness prior to starting treatment.

As AYAs gain awareness the cancer diagnosis is real, and as treatment-related symptom distress begins, they experience times of loneliness and despair. Responses of withdrawing or being ill-tempered because of what is happening to them make it difficult for AYAs to acknowledge or reciprocate HCPs’ efforts to connect. From the beginning, AYAs carefully and continuously assess and monitor HCPs’ actions and behaviours towards them. When they perceive HCPs as unfriendly or uninterested in them, AYAs have little desire to interact with HCPs, resulting in an ongoing sense of *un*connectedness. When AYAs have to regularly interact with HCPs with whom they are unconnected, they feel annoyed and unwilling to engage or communicate.

Parents’ needs to connect with HCPs can also interfere with AYAs’ connectedness. During diagnosis and initial treatment, AYAs experience such high symptom and emotional distress they often do not reciprocate HCP efforts to connect. Parents, on the other hand, are positioned and often eager to form connections with HCPs. Unless AYAs have opportunities to establish their own connections, parents’ connection with HCPs can leave AYAs uncertain about the authenticity of their own connections.

Experiences that foster AYAs’ ability to connect with HCPs include accepting the diagnosis and gaining comfort/familiarity with the environment. When AYAs are ready to connect, they reciprocate HCPs’ efforts by making their own efforts to connect, including acknowledging common bonds, using humour and testing the HCPs trust.

#### Theme categories

We identified four theme categories of the phenomenon. Each theme category is thoroughly explained by describing its subsequent theme clusters that provide detailed descriptions of the participants’ narrative. Theme categories are described using metaphors. Such metaphorical descriptions are occasionally used to enhance the vivid description of participants’ experiences when appropriate. Exemplary quotes were used to support narratives of theme categories and clusters.

#### Theme 1. A cancer diagnosis is like a terrorist attack—a time of traumatic confusion, frustration, fear and vulnerability

Before participants could fully describe their experiences of connectedness with healthcare providers, they talked about the context of the cancer diagnosis. Receiving the diagnosis of cancer is an unexpected time of traumatic confusion, frustration, fear and vulnerability—much like a terrorist attack. The context of the diagnosis sets the stage for connectedness.

##### The timeframe

Like many people who have survived a terrorist attack, participants vividly remember the timeframe and setting during which they received their diagnosis. The moment of the diagnosis seems frozen in time with clear recollections of time-related events surrounding it. Participants precisely recall the date of diagnosis. Robert: “I’ll start from the beginning then. [December 30th, 2013], I was diagnosed with leukaemia. I was diagnosed just after my Christmas vacation. I went back [to school] and noticed I was having a lot of trouble walking around.”

##### A completely unexpected awareness that something might be wrong

Part of the trauma surrounding the participants’ initial diagnosis comes from feeling blindsided by the cancer. Participants consistently described being in situations they thought were safe and comfortable (e.g., in church, at work, at school) when they first recognized something was wrong: “It was [at] a school play … and I felt a lump” [Heather]. Initially, participants brushed off their symptoms and generally did not feel worried, but as symptoms lingered, they became concerned enough to seek medical attention. Robert: “I had problems with my face, it would go numb and … hurt really bad … so, I had to leave school early … to go to the Emergency Room.”

##### Obtaining the diagnosis is difficult and having rude and insensitive HCPS made the situation worse

One particularly frustrating aspect of the participants’ diagnosis experience is the difficulty of finding out what is going on. The difficulty of obtaining a diagnosis of cancer is a difficult experience that is further compounded by experiences of being misdiagnosed or misinformed during the initial consultation. Amy was not given an appropriate diagnosis for several weeks:

I had gone to every major hospital … and they kept telling me I had strep throat … they kept giving me antibiotics … making it worse … Then I went to [the Health Center] … they sent me to get a chest x-ray and they saw a mass in my chest.

There is also an extensive period of time of waiting and worrying before the diagnosis is confirmed.

Another especially frustrating part of the diagnosis occurred when participants interacted with HCPs whom they felt were rude or insensitive. Such encounters created a sense of disconnectedness (i.e., the relationship, or the potential to connect, is destroyed) even before the patient entered the hospital. Julie: “He [the pediatrician] said, ‘Well, there’s usually only one reason you get a lump in your stomach’, implying maybe I was pregnant. At that point, I was like, ‘Okay mom. I’m done talking to him’.” All avenues of communication were shut down from this point forward.

These troubling experiences often left participants with a sense of disconnectedness with HCPs as they entered treatment. This was quite an obstacle that had to be overcome both by the participants and their new paediatric oncology HCPs.

#### Theme 2. Collisions and detours to connectedness—untenable circumstances that hinder the ability to connect

Participants recognize that there are difficult circumstances that hinder both their ability and their willingness to connect with HCPs. The barriers to connectedness are most difficult to overcome at the time of diagnosis. Some of these frustrations are dispelled once participants make sense of their diagnosis, what is happening to them, and come to know the strangers who are trying to help them. However, other challenges remain and continue to prevent participants from connecting with their HCPs. When participants are unable to connect with certain HCPs, they feel unconnected (i.e., having little-to-no common ground upon which to establish any relationship; therefore, no relationship existed) until later, when other opportunities to connect present themselves.

##### Sense of one’s world being turned upside down in a matter of minutes

Much like being involved in a sudden collision and trying to make sense of what happened; the cancer diagnosis is a time when participants try to grasp what is going on. The life-threatening diagnosis of cancer is a sudden, devastating event that interrupts the life route participants were once travelling. Participants experience this moment as a time when, in the midst of coasting along in life, their whole world suddenly turns upside down in a matter of minutes. Participants were so taken aback by the diagnosis it was difficult to comprehend the reality of the situation, especially when information was presented in a matter-of-fact way, as if cancer was normal. Amy: “They [the doctors] took me into a little office and came in and just said ‘I am sorry but you have [cancer] … I was in shock … I thought they were lying.”

##### Loneliness and despair

As the diagnosis became real, sank in, participants experienced a powerful sense of loneliness and despair. Participants are overwhelmed to the point that they are unable to take in HCPs’ efforts to connect. Amy: “I thought … I am going to be a freak and nobody is going to want to talk to me. I just wanted to sleep all of the time. I didn’t want to wake up … I felt sorry for myself.”

##### Feeling trapped in an intolerable environment

Being admitted to a paediatric hospital made participants feel as if they were trapped in an intolerable environment. Amy: “I was 18 and … in a baby hospital … So I am like bored and I want to go home. I missed my friends. There is nothing but babies everywhere, crying.” Ryan: “I hated the Magic Castle people [hospital volunteers]. I really didn’t enjoy their company whatsoever. They would BUST in and be all happy and I’m like ‘Go away!’” The age-inappropriateness of the environment clouded their desire to connect with HCPs.

##### Dark emotions

Participants also described having a strong desire to escape or avoid anything related to their illness and treatments. James: “I hated the hospital … I would have the nurses just keep me knocked out … they were getting on my nerves, so I really didn’t give them the chance.” Participants’ emotional responses, such as grumpiness and irritability, hindered their desire to connect and were perceived by the participants as interfering with HCPs’ willingness to connect with them. Amy: “When they [HCPs] would all meet for rounds, everybody would talk about how they didn’t want to go into my room because I was mean to them.”

##### Treatment-related distress

Because participants received intense cancer treatment regimens, they often felt too sick to talk, which ultimately hindered their ability and/or desire to connect. Chemotherapy side effects and symptom distress caused the most suffering. Ryan: “It [chemotherapy side effects] was just horrible. A lot of throwing up. A lot, for hours. Just dry-heaving and horrible, horrible pain.”

Mood and pain management medications also hindered participants’ ability to feel in control of their behaviour towards some HCPs. Julie: “One time I was on some other drug and it was like … ‘I don’t like you.’ It was terrible. I didn’t know I was doing it.”

##### Sense of being surrounded by strangers who don’t know me

Often, at the scene of a major accident, many strangers gather around trying to help, which can add to the chaos and confusion of the wreck. Similarly, at the beginning of the cancer illness and treatments, participants experience a sense of being surrounded by strangers. Waking up in an unfamiliar and frightening environment, there is a sense of being beset by a myriad of unfamiliar healthcare providers coming in and out of the hospital room. Often this is the participant’s first time being in the hospital. Heather commented:

I was a little scared at first. They put me in isolation, in a room separate by myself for the first time, and I had a lot of people in and out, which was a little awkward for me because I was 16, [a] teenager.

Being surrounded by unfamiliar people, in an unfamiliar environment, contributes to the difficulties in making connections with HCPs.

##### Frustration of the lack of AYA expertise among some HCPS

Participants carefully assessed HCP characteristics during the initial treatment phases. Connectedness was unlikely to occur when participants perceived HCPs as awkwardly intrusive, unfriendly, pushy or unwilling to take time to get to know the AYAs personally. Additionally, connectedness is improbable if HCPs display a limited knowledge of how to take care of and talk to an AYA. Julie: “a lot of the nurses … were used to talking to younger patients. [When they] would come in [and talk to me like a young child], I would think ‘Okay? You talk like that?’” Whenever participants were required to interact with HCPs with whom they did not feel connected, they experienced negative emotional responses such as being annoyed. They also had trouble relating to these HCPs and would shut down all avenues of communication.

#### Theme 3. Parental lenses—parents role in the AYAs’ connectedness with HCPs

Participants experiences of connectedness with HCPs is influenced by the role parents play in their illness and treatment. Because participants are minors at the time of diagnosis, their parents legally must make decisions about treatment and medical care. For participants, when their parents are involved, connectedness experiences are like seeing the world through someone else’s glasses. When healthcare providers speak directly to participants’ parents and leave participants out of the initial opportunity to connect, participants’ perception of their own connection with HCPs is blurred. Initially, participants are too overwhelmed with their diagnosis to participate in much of their own decision-making or healthcare, so they readily allow or expect parents to deal with HCPs and critical decisions. Once participants finally begin to grasp what is happening to them, and have the capability and desire to participate in their own healthcare decisions, their parents are already connected and have become the point persons with HCPs. Although parental connectedness with HCPs does not hinder participants’ abilities to form their own connection with HCPs, it does leave participants with an uncertainty about the possibility and legitimacy of their own connections with HCPs. Such concerns are compounded if participants do not eventually have their own opportunity to form connections.

##### Missed opportunities

Participants experience being left out of initial conversations regarding their diagnosis and/or having few opportunities to meet or connect with some HCPs. Participants who were physically or mentally absent (i.e., participants are in surgery, in a state of shock or asleep) during the initial opportunity to connect with HCPs experience an interference in their abilities to connect with HCPs. Instead of connecting with the participants, HCPs establish connections with participants’ parents. When participants were left out of initial conversations between their parents and HCPs during the diagnosis process, they felt there were few opportunities to meet or connect with some HCPs. Brent: “I think I met … a couple of them [surgeons], maybe even while I was in treatment, but later [my mother told me]; ‘This is Dr. So and So. He did your central line, or your biopsy, or whatever.’” Like wearing someone else’s prescription glasses, connectedness is experienced as a blur or out of focus when participants miss out on the initial opportunity to connect with HCPs, while their parents clearly see the benefits of connecting.

##### Uncertainty about authenticity of connections when parents connect first

When participants miss out on early opportunities to connect, they are unsure of the reality of the connection. In other words, participants are uncertain about the authenticity of their connection to a particular HCP who initially connects with their parent. For example, James described feeling connected to a nurse because of the relationship the nurse had with his mother:

Before I even … started the treatments, I woke up one time and mom was talking to this nurse and [my mother] become real good friends with this nurse, and I hadn’t even met anybody on the floor yet … but they really connected, and throughout my whole stay, when she [the nurse] was on the floor, I was really happy [my] mom was happy because we had someone we would know, and she would come and talk to us and made things a lot better.

In this case, the participant’s connection with the nurse occurred as a result of his appreciation for the nurse who connected with his mother. When parent–HCP connections happened with a specific HCP, participants were less certain about their own connection with that HCP. Once participants began to grasp what was happening to them, they become more ready to initiate connectedness with their HCPs.

#### Theme 4. A game of trust—how connectedness begins

In team building, games such as “If I fall back, will you catch me?” are often played. There is an underlying assumption that others who are playing the game can be trusted enough that one can blindly fall backwards and be caught. Likewise, there is a commitment on behalf of those behind the falling person that they will certainly not let them fall and hit the ground. Similar to this team-building game, connectedness seems to be reciprocal. Participants described specific circumstances that initially foster the desire to connect with healthcare providers.

### Accepting the cancer diagnosis

As participants began to accept having cancer, they realized letting go of their despair fostered their ability and desire to connect. Participants described coming to an acceptance of the illness, understanding the need for help, and being willing to relinquish control as an early and crucial step in allowing oneself to connect. Letting go occurs by gaining an awareness of one’s existential plight, accepting the help being offered, and reconnecting with oneself. Amy:

I just decided I am sick whether I like it or not. I can either take the help they want to give me and fight this, or I can waste the help and die … I didn’t want to die so I had to stop feeling sorry for myself.

### Gaining comfort/familiarity

Acceptance of participants’ illnesses seems to occur when there are opportunities to gain a sense of comfort and familiarity with the hospital, healthcare providers, and treatment routine. Participants do not necessarily recognize familiarity as being part of coming to acceptance; rather, they describe that gaining a sense of familiarity makes them begin to feel more comfortable. For example, Elizabeth described being very intimidated and shy around healthcare providers during her initial diagnosis when she was only in the hospital for a few days; however, when she relapsed and returned to the hospital for an longer period of time, she talked about gaining a sense of familiarity: “It might have been me being there for a while and me realizing … it’s not such a scary place. These people are actually [nice].”

### Strategies AYAs used to connect with HCPs

Once participants were more accepting of having cancer and familiar with the environment and HCPs, they became more receptive to HCPs’ efforts and began initiating their own efforts to connect. Strategies AYAs used included identifying common bonds (i.e., similar personal interests or experiences) and humour. Common interests between participants and HCPs seemed to evoke and deepen connectedness. Brent:

One of them [a nurse] played piano … so when I was in the hospital, and she was in … I’d get really excited because she would take me downstairs to this room with a piano, and we could play duets. That was really fun.

Connectedness was also fostered when HCPs recognized and appreciated participants’ efforts to initiate humor. Amy:

I got these huge glasses with this big huge red nose on it. I put them on, put the blanket over my head, and hit the nurses call button … I said ‘[Dawn], what are the side effects of the morphine? Does it do anything to your nose … because my nose feels really big and it itches’. She was like ‘let me see’ … I lifted the cover and she just busted out laughing.

Lastly, participants described testing whether or not they could trust HCPs. Amy described testing the extent of the oncologist’s trust by covering her head:

when I was feeling sorry for myself, I had my head under the blanket … [Dr. Brown] comes in and he’s like trying to tell us what is going on … My mom is like [Carrie] take that blanket off of your head and listen … [Dr. Brown] said, ’she’s listening’. I was listening. He knew. He knew that I had my mind set, I was feeling sorry for myself right then, but I was still listening.

A sense of connectedness is fostered when HCPs recognize and respond positively to participants’ efforts to connect.

## Discussion

Findings contribute to a better understanding of AYAs’ experiences connecting with HCPs. Four key findings were identified from the essential structure: (1) Diagnosis is a pivotal moment when opportunities for HCPs to connect with AYAs can be missed, lost, or severed if not done delicately. (2) System-wide healthcare efforts to connect with AYAs are not working; participants feel the environment and certain HCPs are teen-unfriendly; (3) Parents’ need to connect with HCPs can inadvertently compete with the AYAs’ opportunities to connect; and (4) AYAs are in the best position to connect when they have had time to accept the diagnosis and gain a sense of familiarity. Following is a discussion of these key findings.

Diagnosis is a pivotal moment when opportunities for connectedness can be missed or severed.

According to participants, the confirmation of the cancer diagnosis is a traumatic moment. This finding relates to three issues that need to be considered when interacting with newly diagnosed AYAs. First, interactions with AYAs are crucial and must be delicately handled, especially around the time of diagnosis. Although AYAs may be unable to take in HCPs’ efforts to connect, they are carefully assessing the characteristics and behaviours of HCPs during this time. This finding makes a unique contribution to the literature because there is little evidence regarding how to establish a relationship with AYAs. Studies that have examined young adult cancer survivors’ perception of communication during diagnosis and treatment support this finding that AYAs are assessing HCPs actions (Zebrack, Chesler, & Kaplan, 2010; Zwaanswijk et al., 2007). Thus, HCPs must pay close attention to how their behaviours and actions may be perceived by AYAs.

The second issue that needs attention is an awareness that AYAs often begin treatment with a sense of disconnectedness with HCPs. Such disconnectedness experiences (i.e., being misdiagnosed, misinformed, or not taken seriously) can negatively influence AYAs’ trust in and perceptions of HCPs in general and make it difficult to connect with their new oncology HCPs. Research supports our finding that AYAs experience delays in diagnosis and often perceive the delivery of the diagnosis as cold and distant (Zebrack, Chesler, & Kaplan, 2010). This important finding should influence the way oncology HCPs conduct initial meetings. Making a genuine effort to repair trust is likely to help minimize disconnectedness and shorten the time it takes for AYAs and HCPs to connect. HCPs can do this by taking time to elicit the AYAs’ perspectives on the events leading up to the diagnosis; using active listening strategies such as “Tell me about your experiences before diagnosis” to encourage AYAs to express their concerns and frustrations; and expressing empathy. HCPs’ display of empathy for past experiences is a central component of patient-provider communication models and have been shown to be effective in fostering connectedness in adult patients (Lin et al., 2014; Matthias & Bair, 2010; Matthias, Salyers, & Frankel, 2013).

The third issue, relevant to connectedness, is HCPs’ awareness of the trauma the AYA experiences with the cancer diagnosis. The adult oncology literature supports the finding that a cancer diagnosis is a traumatic event for patients, and serious psychological consequences can result if HCPs do not appropriately handle interactions at this time (Paul, Clinton-McHarg, Sanson-Fisher, Douglas, & Webb, 2009). HCPs can have a profound impact on how patients cope with their diagnosis. A theoretical model that demonstrates the potential impact that HCPs have on AYA patients is the Resilience in Illness Model (formerly called the Adolescent Resilience Model) (Haase, 2004; Haase, Kintner, Monahan, & Robb, 2014). In this model, the nature of the AYA–HCP relationship is a protective factor and considered to have an influence on moving AYAs away from the use to defensive coping strategies and towards courageous coping strategies. Although defensive ways of coping are initially used for protection in life-threatening situations, they become problematic when they are sustained and prevent the development of more positive ways of coping (Haase, 2004). Therefore, if HCPs make early efforts to connect with AYAs and display an acceptance of the use of defensive coping strategies, then AYAs may be more likely to also use courageous coping strategies, especially supportant coping, which is defined as the willingness to reach out and ask for help/support when needed (Haase, 2004). This ultimately helps foster resilience and enhanced well-being.

If positive efforts to connect with AYAs are not effectively initiated, the opportunity to connect may be missed, resulting in unconnectedness (i.e., no relationship), which, in turn, can hinder further open communication between the AYAs and HCPs. Persistent lack of open communication could have a negative impact on AYAs’ decision making and willingness to engage in long-term follow-up (Haase, 2004; Zebrack et al., 2010). Therefore, the diagnosis and initial treatment phase is a key time when HCPs need to be attuned to how their interactions and behaviours are interpreted by AYAs.

### System-wide healthcare efforts to connect with AYA are not working

The lack of AYA-appropriate health services is recognized as a major problem in the United States. To address the nation’s deficit in providing adequate health services to AYA, the National Research Council and the Institute of Medicine, along with the Board of Children, Youth, and Families, formed the Committee on Adolescent Health Services and Models of Care for Treatment, Prevention, and Healthy Development. According to the Committee on Adolescent Health Services (2009),

the health system—health services, the settings where these services are delivered … has an important role to play in promoting healthful behavior, managing health conditions, and preventing disease during adolescence. Yet health services and settings in the United States today are not designed to help young people at this critical time in their lives, and providers often are not adequately trained in adolescent issues. As is the case in many other parts of the nation’s health system, adolescents face gaps in care, fragmented services, and missed opportunities for health promotion and disease prevention. (p. 1)

Similarly, in the field of AYA oncology, there is an increased focus on ways to better meet the needs of AYAs with cancer (Wilkins, D’Agostino, Penney, Barr, & Nathan, 2014). Although researchers have acknowledged that AYAs have unique needs that differ from their younger or older counterparts, there is little empirical evidence on how to best support the needs of AYAs undergoing cancer treatment (Adolescent and Young Adult Oncology Progress Review Group (AYAO PRG), 2006; Wilkins et al., 2014). One of the reasons that it may be difficult to examine standards of practice that best support the needs of AYA is that currently there is no consensus on where AYAs should be treated. Depending on their diagnoses and means of referral, AYA are either treated in a paediatric or adult oncology setting (Bleyer, 2007). In either setting, AYAs have expressed feeling misplaced (Zebrack et al., 2009).

Researchers in the United Kingdom found that environments that provides specialized cancer care to AYAs may have a positive influence on health outcomes of AYAs with cancer (Kelly, Pearce, & Mulhall, 2004; Mulhall, Kelly, & Pearce, 2004; Whelan, 2003). In the UK, Teenage Cancer Units are specialized units created for AYAs and equipped with computers with internet access, web cams, video games, musical instruments and DVDs. There are also lounges for AYAs to just relax or visit with family and friends. HCPs who work on these units receive special training in how to communicate and care for AYAs. Currently, there are eight teenage cancer units in the UK. Although only two descriptive studies were found to have evaluated a specialized AYA cancer unit (Kelly et al., 2004; Mulhall et al., 2004), results indicate that both AYAs and parents highly valued and appreciated the teen-friendliness of the environment as well as the staff’s sensitivity towards teenagers. However, there seems to be some hesitancy of implementing AYA cancer units in other countries. Reasons for this hesitancy include the cost-effectiveness of developing a specialized unit for such a small number of patients and difficulty of coordinating the care for AYAs in one specific area who are followed by paediatric oncologists versus adult oncologists (Whelan, 2003). As an alternative to specialized AYA units, research should focus on identifying practical ways HCPs and hospital environments can become more AYA-centered and on developing and evaluating AYA communication training programmes for HCPs.

### Parents’ need to connect with HCPs can inadvertently compete with the AYAs’ opportunities to connect

Although parents play an instrumental role in supporting and assuming primary responsibility for their AYAs’ healthcare decisions, their own need to connect with HCPs may inadvertently compete with the AYAs’ opportunities to connect. If AYAs are unable to later develop their own connection with HCPs, it could interfere with their ability to assume responsibility for their own health during and after treatment. Other studies of AYAs with chronic conditions have found the more involved parents are in care, the less control and interest the adolescents have in their own disease management (Huang et al., 2011). The potential interference of parents in AYA–provider connectedness could be one of the reasons AYA cancer survivors are poor consumers of healthcare and engage in risky behaviours that jeopardize their health (Institute of Medicine & National Research Council, 2003). Further research is needed to examine the influence of parent–provider connectedness on AYA healthcare self-management.

### AYAs are in the best position to connect when they have had time to accept the diagnosis and gain a sense of familiarity

To foster their ability to connect with HCPs, AYAs need help accepting their illness and gaining familiarity with the environment. Our findings indicate AYAs’ process of accepting their illness cannot be rushed; making consistent efforts to familiarize AYAs with the environment, people and routine may help. To assist AYAs to navigate the cancer experience early and reduce the time needed for AYAs and HCPs to connect, we recommend developing a computerized psychosocial assessment tool, so that AYAs who don’t want to talk or having trouble expressing their thought can still communicate. Such an assessment would be used as an evidence-based way to introduce the AYA to the healthcare team.

When AYAs are ready to make efforts to connect, HCPs need to sensitively acknowledge and foster these efforts. Engaging AYAs in conversations about their personal interests and things AYAs find humorous are suggested strategies. In addition, because shared experiences solidify connectedness, based on what HCPs learn about AYAs’ interests, they should consider sharing something personal about themselves (i.e., common interests in particular activities, hobbies or events) (Phillips-Salimi, Haase, & Kooken, 2012; Ventres & Frankel, 2015).

#### Study strengths and limitations

This study has strengths and limitations. The first strength is that this study is one of the first studies to have examined the lived experiences of connectedness with HCPs from the perspective of AYAs. Secondly, the study sample had a good representation of gender and the most common cancer types seen in the AYA population; however, there could have been a better representation of race. Limitations, however, included a small sample size, implying that the findings cannot be generalized to all AYAs. Lastly, participants were all actively engaged in long-term cancer follow-up and felt connected with their HCPs. AYAs who were not engaging in long-term follow-up may have had different experiences. Despite these shortcomings, this study provides an initial understanding of AYA experiences to connecting with HCPs.

## Conclusion

This study explored the lived experience of AYAs connecting with HCPs. Our findings revealed that AYAs’ experiences of connecting with HCPs are delicate experiences that can be complicated by several factors, especially prior to the cancer diagnosis. A commonality among the participants was starting their experience of connecting with HCPs from a disconnectedness perspective. Future research should focus on key strategies AYAs perceive as important in fostering connectedness and identifying practical ways HCPs and the healthcare environment can become more AYA-friendly. This study contributes to the body of knowledge regarding the lived experience of AYAs connecting with their HCPs. Additionally, the identified factors that can hinder connectedness, such as previous experiences of disconnectedness, unfamiliarity and sense of overwhelmness, can be used to help develop interventions to establish early connectedness; ultimately improving the health and well-being of AYAs during the cancer experience.

## References

[CIT0001] Adolescent and Young Adult Oncology Progress Review Group (AYAO PRG) (2006). Closing the gap: Research and care imperatives for adolescents and young adults with cancer. Retrieved 3, 25, 2015, from http://planning.cancer.gov/library/AYAO_PRG_Report_2006_FINAL.pdf

[CIT0002] Beach M. C., Keruly J., & Moore R. D. (2006). Is the quality of the patient-provider relationship associated with better adherence and health outcomes for patients with HIV? *Journal of General Internal Medicine*, 21(6), 661–10. doi:10.1111/j.1525-1497.2006.00399.x 16808754 PMC1924639

[CIT0003] Bleyer A. (2007). Adolescent and young adult (AYA) oncology: The first A. *Pediatric Hematology and Oncology*, 24(5), 325–336. doi:10.1080/08880010701316850 17613877

[CIT0004] Bleyer, A., Ferrari, A., Whelan, J., & Barr, R. D., (2017) Global assessment of cancer incidence and survival in adolescents and young adults. *Pediatric Blood & Cancer*, doi:10.1002/pbc.26497

[CIT0005] Brion J. (2014). The patient-provider relationship as experienced by a diverse sample of highly adherent HIV-infected people. *The Journal of the Association of Nurses in AIDS Care : JANAC*, 25(2), 123–134. doi:10.1016/j.jana.2013.01.006 23809659

[CIT0006] Colaizzi P. F. (1978). Psychological research as the phenomenologist views it In Valle R. & King M. (Eds.), *Existential-phenomenological alternatives for psychology* (pp. 48–71). New York: Oxford University Press.

[CIT0007] Committee on Adolescent Health Care Services and Models of Care for Treatment, Prevention, and Healthy Development (2009). *Adolescent health services: Missed opportunities*. Washington, DC: The National Academies Press.

[CIT0008] Cresswell J. W. (1998). *Qualitative inquiry and research design: Choosing among five traditions*. Thousand Oaks, CA: Sage.

[CIT0009] Diaz V. A., Mainous A. G. 3rd, Gavin J. K., Player M. S., & Wright R. U. Jr. (2015). Use of a tablet-based risk assessment program to improve health counseling and patient-provider relationships in a federally qualified health center. *American Journal of Medical Quality*. doi:10.1177/1062860615587012 25995332

[CIT0010] Earle E. A., Davies H., Greenfield D., Ross R., & Eiser C. (2005). Follow-up care for childhood cancer survivors: A focus group analysis. *European Journal of Cancer*, 41(18), 2882–2886. S0959-8049(05)00837-3. doi:10.1016/j.ejca.2005.08.028 16275059

[CIT0011] Giorgi A. (2005). The phenomenological movement and research in the human sciences. *Nursing Science Quarterly*, 18(1), 75–82. doi:10.1177/0894318404272112 15574702

[CIT0012] Guba E. G., & Lincoln Y. S. (1981). *Effective evaluation: Improving the usefulness of evaluation results through responsive and naturalistic approaches*. San Franciso: Jossey-Bass.

[CIT0013] Haase J. E. (1987). Components of courage in chronically ill adolescents: A phenomenological study. *ANS. Advances in Nursing Science*, 9(2), 64–80. 3099638 10.1097/00012272-198701000-00010

[CIT0014] Haase J. E. (2004). The adolescent resilience model as a guide to interventions. *Journal of Pediatric Oncology Nursing*, 21(5), 289–299. doi:10.1177/104345420426792221/5/289 15381798

[CIT0015] Haase J. E., Kintner E. K., Monahan P. O., & Robb S. L. (2014). The resilience in illness model, part 1: Exploratory evaluation in adolescents and young adults with cancer. *Cancer Nursing*, 37(3), E1–12. doi:10.1097/NCC.0b013e31828941bb PMC3758400 23519038

[CIT0016] Huang J. S., Gottschalk M., Pian M., Dillon L., Barajas D., & Bartholomew L. K. (2011). Transition to adult care: Systematic assessment of adolescents with chronic illnesses and their medical teams. *The Journal of Pediatrics*, 159(6), 994–998 e992. doi:10.1016/j.jpeds.2011.05.038 21784450 PMC3215794

[CIT0017] Husserl E. (1970). *Crisis of european sciences and transcendental phenomenology*. (D. Carr, Trans.) Chicago, IL: Northwestern University Press.

[CIT0018] Institute of Medicine (IOM) (2013). Identifying and addressing the needs of adolescents and young adults with cancer - Workshop summary. *Accessed March*, 25, 2015 Available at http://iom.edu/Reports/2013/Identifying-and-Addressing-the-Needs-of-Adolescents-and-Young-Adults-with-Cancer.aspx

[CIT0019] Institute of Medicine, & National Research Council (2003). *Childhood cancer survivorship: Improving care and quality of life*. Washington, D.C.: National Academy of Sciences.

[CIT0020] Kelly D., Pearce S., & Mulhall A. (2004). ‘Being in the same boat’: Ethnographic insights into an adolescent cancer unit. *International Journal of Nursing Studies*, 41(8), 847–857. doi:10.1016/j.ijnurstu.2004.03.011 15476758

[CIT0021] Leidy N. K., & Haase J. E. (1996). Functional performance in people with chronic obstructive pulmonary disease: A qualitative analysis. *ANS. Advances in Nursing Science*, 18(3), 77–89. 8660014 10.1097/00012272-199603000-00008

[CIT0022] Lin J. J., Lake J., Wall M. M., Berman A. R., Salazar-Schicchi J., Powell C., … Wisnivesky J. P. (2014). Association of patient-provider communication domains with lung cancer treatment. *Journal of Thoracic Oncology*, 9(9), 1249–1254. doi:10.1097/JTO.0000000000000281 25122421 PMC4133738

[CIT0023] Marelich W. D., & Murphy D. A. (2003). Effects of empowerment among HIV-positive women on the patient-provider relationship. *AIDS Care*, 15(4), 475–481. 14509862 10.1080/0954012031000134719

[CIT0024] Matthias M. S., & Bair M. J. (2010). The patient-provider relationship in chronic pain management: Where do we go from here? *Pain Medicine*, 11(12), 1747–1749. doi:10.1111/j.1526-4637.2010.00998.x 21134115

[CIT0025] Matthias M. S., Salyers M. P., & Frankel R. M. (2013). Re-thinking shared decision-making: Context matters. *Patient Education and Counseling*, 91(2), 176–179. doi:10.1016/j.pec.2013.01.006 23410979

[CIT0026] Mulhall A., Kelly D., & Pearce S. (2004). A qualitative evaluation of an adolescent cancer unit. *European Journal of Cancer Care*, 13(1), 16–22. doi:10.1111/j.1365-2354.2003.00434.x 14961771

[CIT0027] Nass S. J., Beaupin L. K., Demark-Wahnefried W., Fasciano K., Ganz P. A., Hayes-Lattin B., … Smith A. W. (2015). Identifying and addressing the needs of adolescents and young adults with cancer: Summary of an Institute of Medicine workshop. *The Oncologist*, 20(2), 186–195. doi:10.1634/theoncologist.2014-0265 25568146 PMC4319626

[CIT0028] Oeffinger K. C., Mertens A. C., Hudson M. M., Gurney J. G., Casillas J., Chen H., … Robison L. L. (2004). Health care of young adult survivors of childhood cancer: A report from the Childhood Cancer Survivor Study. *Annals of Family Medicine*, 2(1), 61–70. doi:10.1370/afm.26 15053285 PMC1466633

[CIT0029] Oeffinger, K. C., Mertens, A. C., Sklar, C. A., Kawashima, T., Hudson, M. M., Meadows, A. T., … Robison, L. L. (2006). Chronic health conditions in adult survivors of childhood cancer. The New England Journal of Medicine, 355(15),1572–1582. 355/15/1572. doi:10.1056/NEJMsa060185

[CIT0030] Oeffinger K. C., & Wallace W. H. (2006). Barriers to follow-up care of survivors in the United States and the United Kingdom. *Pediatric Blood & Cancer*, 46(2), 135–142. doi:10.1002/pbc.20614 16369921

[CIT0031] Paul C. L., Clinton-McHarg T., Sanson-Fisher R. W., Douglas H., & Webb G. (2009). Are we there yet? The state of the evidence base for guidelines on breaking bad news to cancer patients. *European Journal of Cancer*, 45, 2960–2966. doi:10.1016/j.ejca.2009.08.013 19762227

[CIT0032] Phillips-Salimi C. R., Haase J. E., & Kooken W. C. (2012). Connectedness in the context of patient-provider relationships: A concept analysis. *Journal of Advanced Nursing*, 68(1), 230–245. doi:10.1111/j.1365-2648.2011.05763.x 21771040 PMC3601779

[CIT0033] Phillips-Salimi C. R., Lommel K., & Andrykowski M. A. (2012). Physical and mental health status and health behaviors of childhood cancer survivors: Findings from the 2009 BRFSS survey. *Pediatric Blood & Cancer*, 58(6), 964–970. doi:10.1002/pbc.23359 22012636 PMC3332525

[CIT0034] Recklitis C. J., Lockwood R. A., Rothwell M. A., & Diller L. R. (2006). Suicidal ideation and attempts in adult survivors of childhood cancer. *Journal of Clinical Oncology*, 24(24), 3852–3857. doi:10.1200/JCO.2006.06.5409 16921037

[CIT0035] Sandelowski M. (1986). The problem of rigor in qualitative research. *Advances in Nursing Science*, 8(3), 27–37. 3083765 10.1097/00012272-198604000-00005

[CIT0036] Sheppard V. B., Adams I. F., Lamdan R., & Taylor K. L. (2011). The role of patient-provider communication for black women making decisions about breast cancer treatment. *Psycho-Oncology*, 20(12), 1309–1316. doi:10.1002/pon.1852 20941804

[CIT0037] Tai E., Buchanan N., Townsend J., Fairley T., Moore A., & Richardson L. C. (2012). Health status of adolescent and young adult cancer survivors. *Cancer*, 118(19), 4884–4891. doi:10.1002/cncr.27445 22688896 PMC5292773

[CIT0038] Ventres W. B., & Frankel R. M. (2015). Shared presence in physician-patient communication: A graphic representation. *Families, Systems & Health*, 33(3), 270–279. doi:10.1037/fsh0000123 26075882

[CIT0039] Whelan J. (2003). Where should teenagers with cancer be treated? *European Journal of Cancer*, 39(18), 2573–2578. 14642919 10.1016/j.ejca.2003.09.014

[CIT0040] Wilkins K. L., D’Agostino N., Penney A. M., Barr R. D., & Nathan P. C. (2014). Supporting adolescents and young adults with cancer through transitions: Position statement from the Canadian Task Force on Adolescents and Young Adults with cancer. *Journal of Pediatric Hematology/Oncology*, 36(7), 545–551. PMID: 24390448. doi:10.1097/MPH.0000000000000103 24390448

[CIT0041] Zebrack B., Chesler M. A., & Kaplan S. (2010). To foster healing among adolescents and young adults with cancer: What helps? What hurts? *Supportive Care in Cancer*, 18(1), 131–135. doi:10.1007/s00520-009-0719-y 19690897

[CIT0042] Zwaanswijk M., Tates K., van Dulmen S., Hoogerbrugge P. M., Kamps W. A., & Bensing J. M. (2007). Young patients’, parents’, and survivors’ communication preferences in paediatric oncology: Results of online focus groups. *BMC Pediatrics*, 7, 35. doi:10.1186/1471-2431-7-35 17996108 PMC2206018

